# Metabolomics and triple-negative breast cancer: A systematic review

**DOI:** 10.1016/j.heliyon.2023.e23628

**Published:** 2023-12-13

**Authors:** Meritxell Arenas, Maria Fargas-Saladié, Marta Moreno-Solé, Lucía Moyano-Femenia, Andrea Jiménez-Franco, Marta Canela-Capdevila, Helena Castañé, Cristian Martínez-Navidad, Jordi Camps, Jorge Joven

**Affiliations:** a Department of Radiation Oncology, Hospital Universitari Sant Joan de Reus, Institut D'Investigació Sanitària Pere Virgili, Universitat Rovira I Virgili, Reus, Spain; b Unitat de Recerca Biomèdica, Hospital Universitari Sant Joan de Reus, Institut D'Investigació Sanitària Pere Virgili, Universitat Rovira I Virgili, Reus, Spain

**Keywords:** Biomarkers, Breast cancer, Metabolomics, Neoadjuvant therapy, Therapeutic targets

## Abstract

Triple-negative breast cancer stands out as the most aggressive subtype of breast malignancy and is characterized by an unfavourable prognosis. *Objective*: This systematic review summarizes the insights gleaned from metabolomic analyses of individuals afflicted with this cancer variant. The overarching goal was to delineate the molecular alterations associated with triple-negative breast cancer, pinpointing potential therapeutic targets and novel biomarkers. *Methods:* We systematically searched for evidence using the PubMed database and followed the PRISMA and STARLITE guidelines. The search parameters were delimited to articles published within the last 13 years. *Results:* From an initial pool of 148 scrutinized articles, 17 studies involving 1686 participants were deemed eligible for inclusion. The current body of research shows a paucity of studies, and the available evidence presents conflicting outcomes. Notwithstanding, Pathway Enrichment Analysis identified the urea and glucose-alanine cycles as the most affected metabolic pathways, followed by arginine, proline, and aspartate metabolism. *Conclusion:* Future investigations need to focus on elucidating which of those metabolites and/or pathways might be reliable candidates for novel therapeutic interventions or reliable biomarkers for diagnosis and prognosis of this subtype of breast cancer.

## Highlights

• Triple-negative is the most aggressive subtype of breast cancer.
• This is a systematic review of metabolic alterations in this neoplasm.
• We identified 9 upregulated and 3 downregulated metabolites.
• They were related to energy metabolism and associated pathways.

## 1 Introduction

Breast cancer (BC) is the most prevalent solid cancer in women and the second leading cause of malignant neoplasm-related mortality [1]. The management of BC is complex because of the considerable molecular heterogeneity exhibited by tumours, which has implications for the risk of recurrence and response to therapeutic interventions [2]. To date, several distinct BC phenotypes have been recognized, namely luminal A, luminal B, human epidermal growth factor 2 receptor (HER2)-positive, and triple-negative, based on the presence or absence of specific molecular targets, including estrogen receptors (ER), progesterone receptors, HER2, and Ki-67 antigen expression levels. The absence of these molecular targets typifies triple-negative breast carcinoma (TNBC), the most aggressive subtype. Indeed, patients under 40 with TNBC exhibit an early recurrence hazard, frequent visceral and brain metastases, and a diminished survival rate compared to other subtypes [3,4]. In this framework, substantial effort has been directed towards scrutinizing the underlying factors that contribute to the development of treatment resistance and progression of TNBC. Finding biomarkers will help to define personalized treatments, thus enhancing the quality of life and overall survival of affected patients [5].

To date, neoadjuvant chemotherapy (NAC) is the preferred treatment for TNBC [[6], [7], [8]]. Complete pathological response to NAC is associated with improved disease-free survival. Neoadjuvant regimens involving anthracyclines and taxanes or taxanes with an alkylating agent are standard for treating stage II or III TNBC patients, followed by capecitabine for those in whom the disease remains [9]. Moreover, new treatments, such as olaparib, talazoparib, and pembrolizumab, have recently been approved [10]. Regrettably, treatment outcomes vary, with some patients manifesting a robust and completely favourable pathological response but others only partial improvement. In certain cases, the disease may stabilize or progress. The underlying reasons for these disparate treatment responses still need to be explained and better understood.

TNBC's lack of hormone and gene receptor positivity eliminates many therapeutic possibilities and contributes to its aggressive and lethal nature. Therefore, there is a critical need for new research to identify molecules that can be targeted for therapy or serve as prognostic biomarkers [11]. A line of investigation of increasing interest in the scientific community is the metabolic alterations that cancer produces. Malignant cells undergo substantial metabolic modifications compared to healthy cells [12]. This reprogramming facilitates the provision of metabolites essential for lipid, protein, and nucleic acid biosynthesis, which fosters tumour cell proliferation. We hypothesize that investigating the metabolic alterations in blood and tumour tissue of TNBC patients may contribute significantly to understanding this disease. This systematic review aims to summarize the progress made through metabolomic investigation in delineating modifications within the tumour tissue or systemic circulation of women afflicted with TNBC. The primary goal is to identify candidate biomarkers and therapeutic targets arising from these metabolic modifications. Furthermore, our analysis extends to evaluating whether the expression patterns of specific metabolites correlate or not with the response to NAC.

## 2 Methods

### 2.1 Search strategy and eligibility criteria

We systematically identified relevant literature following the PRISMA and STARLITE statements. Initially, we used an expert chain-of-citations method to conduct a comprehensive search. Subsequently, we utilized a computerized search strategy based on specific keywords and implemented publication date limits. Considering the search criteria, we anticipated that English-language publications would sufficiently cover pertinent non-English-language articles. We used an overlapping subsets technique and utilized the following Boolean phrase within the Pubmed database: (“METABOLOMICS” AND “TRIPLE NEGATIVE BREAST CANCER”). The inclusion criteria were predefined, and the protocol was restricted to the following specifications.

· Articles published within the last 13 years, from January 1, 2010, to January 1, 2023.
· Articles in English.
· Observational studies involving human subjects.
· Studies conducted exclusively on women.
· Studies including at least one group of patients with TNBC.
· We excluded studies conducted in non-human models (*in vitro* or xenograft models). Additionally, we disregarded incomplete studies which lacked specific sample sizes or results. Studies that did not primarily analyse metabolomics or were not centred mainly on TNBC were also excluded. No geographic or ethnic restrictions were applied in the inclusion or exclusion criteria.

### 2.2 Selection criteria

The primary focus of the selection process was to identify metabolites that exhibited differential regulation in TNBC compared to other types of BC or healthy individuals. Furthermore, we considered articles that explored metabolites associated with predicting treatment outcomes in TNBC and those linked to a higher risk of metastasis.

**Fig. 1:**
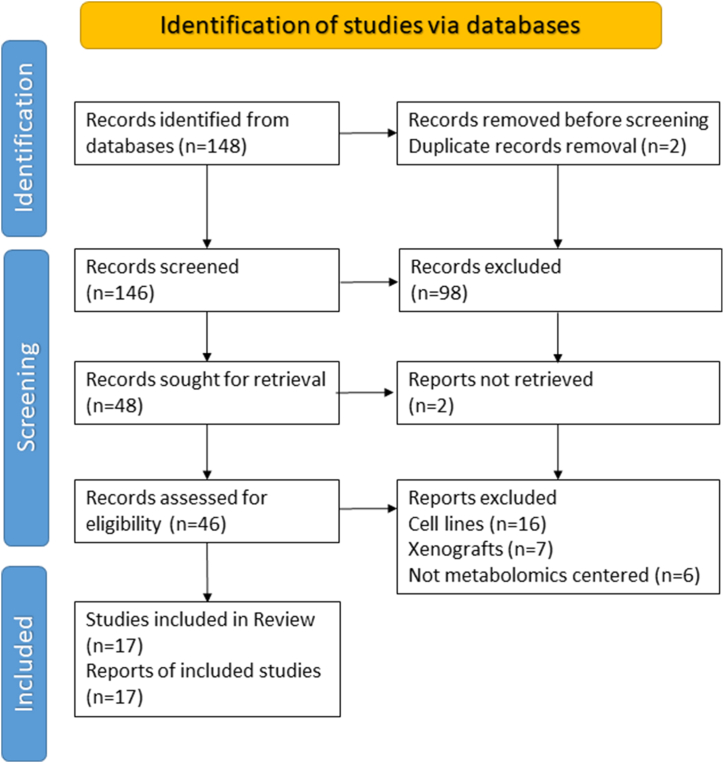
A flow diagram of Preferred Reporting Items for Systematic Reviews and Meta-Analyses (PRISMA).

The initial electronic search performed in PubMed yielded 148 articles (Fig. 1). Two papers were immediately excluded by the automation tool Mendeley, with one being ineligible due to consisting solely of a series of abstracts without further analysis and the other being a duplicate. Subsequently, the remaining 146 articles underwent abstract screening. Ninety-eight records did not satisfy our selection criteria, and 46 full-text articles were assessed by our team for eligibility. Finally, 17 manuscripts met the inclusion criteria for this systematic review. Three researchers independently reviewed all the articles. Most reports adopted a case-control study design (14 studies), while three were cohort studies. In total, 1686 participants were included in the analyses. Among these, five articles focused exclusively on investigating metabolites with higher levels in TNBC, compared with either other types of BC or healthy subjects. Conversely, none of the studies examined solely those metabolites with lower levels in TNBC. Nine studies analysed both higher and lower levels. Additionally, three articles identified metabolites in TNBC that showed associations with a poorer response to NAC.

### 2.3 Analysis of the results

The large number of metabolites analysed by the various authors and the diverse technologies used made interpreting the results a notable challenge. We overcame that by including those metabolites identified in more than one study. We used Pathway Enrichment Analysis in MetaboAnalyst 5.0 (https://www.metaboanalyst.ca/).

## 3 Results

We identified 139 metabolite levels as higher, 77 as lower, and 26 as associated with poor NAC responses (Supplementary Table 1). The following subsections report an individualized analysis of the 13 metabolites that had been identified in more than one article, either in tumour samples or in circulation. These results are also summarized in Table 1.

**Table 1:** Metabolites reported in more than one article.

Metabolite	Number of articles	Sample	Reference number	Type of change
Creatine	4	Tumour Tumour Tumour Serum	[13] [14] [15] [16]	Increase Increase Decrease Increase
Glutamate	4	Tumour Tumour Tumour Serum	[13] [15] [17] [18]	Increase Increase Increase Decrease
Choline	3	Tumour Tumour Tumour	[13] [15] [19]	Increase Increase Increase
Proline	3	Plasma Plasma Plasma	[16] [20] [21]	Increase Increase Decrease
Leucine	3	Tumour Tumour Serum	[13] [16] [22]	Increase Increase Decrease
Isoleucine	2	Tumour Serum	[13] [22]	Increase Decrease
Glucose	2	Tumour Serum	[14] [18]	Increase Increase
Citrulline	2	Tumour Tumour	[13] [19]	Increase Increase
Diacetyl spermine	2	Plasma Plasma	[23] [24]	Increase Increase
Kynurenine	2	Tumour Tumour and plasma	[13] [25]	Increase Increase
Tyrosine and alanine	2	Plasma Plasma	[18] [21]	Decrease Decrease
Glutamine	2	Tumour Tumour	[13] [15]	Decrease Decrease

This table shows the list of metabolites found with significantly modified levels in more than one article in patients with triple-negative breast cancer, compared to healthy controls or other cancer types, and in tissue samples or the circulation.

### 3.1 Creatine

Creatine levels in tumour tissue were analysed in three articles. Kanaan et al. [13] conducted a study involving biopsies from TNBC (n = 15) and ER-positive (n = 15) patients obtained from 30 African-American women. These samples were collected before administering any chemotherapy, hormonal treatment, or radiotherapy. The study identified 418 metabolites, of which only 133 (31.8 %) showed significant differences. Creatine concentrations were higher in TNBC samples, and the authors suggested that this reflected the heightened amino acid uptake and the metabolic activity associated with TNBC cells. Tayyari et al. [14] investigated tumour samples obtained from 47 patients, 18 with TNBC and 29 with Luminal A. The study compared the characteristics of affected tissue with adjacent normal tissue. They observed a significant increase in creatine levels in affected tissue of TNBC patients compared to normal tissue. In contrast, no such elevation was observed in Luminal A samples. Cao et al. [15] characterized distinct metabolic profiles in TNBC through metabolomic identification in tumour biopsies obtained from 75 BCE patients with no known metastases. In contrast to the findings of previous authors, they found significantly lower creatine levels in TNBC tumours compared to HER2/ER-positive samples.

Serum creatine levels were analysed in one article. Li et al. [16] reported elevated creatine levels in 31 TNBC patients compared to 31 healthy controls. The consistent increase in amino acid levels observed in that study suggested an alteration in the aminoacyl-tRNA biosynthesis pathway, which is crucial for protein synthesis and cellular viability. The authors postulated that the increased demand for protein synthesis in TNBC could be attributed to the substantial biomass required for tumour growth.

### 3.2 Glutamate

Glutamate plays a role in glutathione biosynthesis, amino acid biosynthesis, and DNA methylation. Changes in tumour biopsies referencing these functions were documented in three articles. In the above-mentioned study, Kanaan et al. [13] observed significantly higher glutamate levels in TNBC tumours compared to ER-positive tumours. Cao et al. [15] found higher levels of glutamate and lower levels of glutamine in TNBC tumours compared to HER2/ER-positive samples. Dragan et al. [17] investigated breast biopsies and plasma from 79 individuals, 52 with TNBC and 26 healthy controls. They proposed that overexpression of the KISS1R gene leads to significantly higher glutamate levels in primary tumours than in control tumours. This effect might be attributed to the enhanced utilization of serum glutamine for glutaminolysis, resulting in higher glutamate content. The study also demonstrated a larger glutamate pool in cells overexpressing KISS1R than in controls. Moreover, metabolomic analyses of human primary tumour biopsies revealed higher glutamate levels in TNBC compared to ER + tumours.

Wojtowicz et al. [18] reported lower plasma glutamate concentrations in a study comparing plasma samples from 9 TNBC patients and 86 healthy controls. Those results were further validated by studying the metabolomic changes in the culture medium MDA-MB-468, an *in vitro* model of TNBC.

### 3.3 Choline

Choline changes in tumour tissue were referenced in three articles. Kanaan et al. [13] observed a significant elevation in choline levels in TNBC compared to ER-positive BC. Cao et al. [15] corroborated those findings. Choline is crucial in lipid metabolism, cell membrane synthesis and degradation, and cell signalling. The authors proposed that high choline levels contribute to greater glycolytic activity, which is directly associated with increased tumour aggressiveness. Yamashita et al. [19] conducted a study involving 72 BCE tissue samples (11 of which belonged to TNBC patients) and corresponding adjacent cancer-free tissues. They found significantly higher in choline concentrations in TNBC samples and slightly elevated phosphocholine levels compared with hormone receptor-positive tumours. Immunostaining for choline kinase did not reveal substantial differences between them.

### 3.4 Proline

Circulating proline levels in TNBC are discussed in three articles. However, there is conflicting evidence, with two studies suggesting high concentrations of this amino acid being present [16,20] and one indicating low concentrations [21]. Li et al. [16] found 77 significantly different metabolites between BC patients and controls. Proline levels were higher in all subtypes, particularly in TNBC. He et al. [20] observed higher proline levels in the serum of patients who showed a poor response to NAC, defined as a reduction in tumour volume of less than 30 % or an increase of no more than 20 %. The study included 52 TNBC patients; samples were collected before and after NAC administration. Changes in tumour volume were assessed using various imaging techniques and histopathology. According to the response, patients were classified into three subgroups: poor or no response, partial response, and complete response. Proline allowed differentiation from the complete response subgroup, where levels were the lowest. In contrast to the above findings, Shen et al. [21] examined plasma samples from 60 women with BC and 60 healthy controls, and they observed lower levels of most amino acids in BC patients with respect to the controls, especially those with TNBC. Among the ten amino acids that showed significantly lower TNBC levels than controls, eight remained statistically significant after adjusting for multiple comparisons, including proline, tyrosine, and alanine.

### 3.5 Leucine and isoleucine

Leucine in tumour biopsies is referenced in two articles, while isoleucine is mentioned in one. Kanaan et al. [13] reported that tumour leucine and isoleucine levels were both significantly higher in TNBC than in ER-positive BC. Li et al. [16] discussed the dysregulation of leucine in the aminoacyl-tRNA biosynthesis pathway. That study found significantly higher leucine levels in TNBC patients compared to healthy volunteers.

Serum leucine and isoleucine concentrations were studied by Arenas et al. [22], which involved 195 individuals, of whom 151 were BC patients (18 with TNBC) and 44 were healthy controls. The study aimed to investigate metabolic changes associated with energy balance during radiotherapy. After surgery and radiotherapy, alterations in serum concentrations of glycolysis products, citric acid cycle intermediates, and amino acid metabolism were reported. Among these, leucine alterations stood out, being lower in all BC serums. Following irradiation, leucine and isoleucine concentrations normalized and became comparable to the control groups.

### 3.6 Glucose

Glucose levels in tumours is mentioned in one article. Tayyari et al. [14] demonstrated that glucose levels were significantly higher in affected tissues of TNBC patients compared to adjacent normal tissue. Plasma glucose concentrations were also found to be higher by Wojtowicz et al. [18] in the study mentioned above.

### 3.7 Citrulline

Changes in citrulline levels in tumour tissue are mentioned in two articles. Kanaan et al. [13] showed that TNBC has elevated tumour arginine levels and its intermediates, including citrulline. These findings suggested alterations in multiple pathways associated with higher amino acid uptake and protein catabolism. Citrulline and other components of the urea cycle showed significant increases, leading to greater pro-inflammatory signalling. Yamashita et al. [19] analysed metabolites related to the urea cycle and observed significant elevations in aspartate, arginine, and citrulline levels in tissue samples.

### 3.8 Diacetyl spermine

Diacetyl spermine (DAS), a catabolic product of spermine mediated through tumour spermine synthase, was analysed in plasma in two articles. Irajizad et al. [23] conducted a study involving 255 patients, 88 with TNBC, and 167 controls. Higher plasma DAS levels were found in TNBC patients with poor disease-free survival and overall survival. Plasma DAS levels better discriminated TNBC cases from controls. Fahrmann et al. [24] analysed plasma samples from newly diagnosed TNBC patients and cancer-free controls. They observed higher plasma DAS associated with greater metastatic activity. Additionally, mRNA expression of spermine synthase in TNBC tumour tissue predicted poor overall survival and a higher risk of distant metastasis.

### 3.9 Kynurenine

Kynurenine is a metabolite of the amino acid l-tryptophan used to produce niacin and is mentioned in two articles. Kanaan et al. [13] postulated that TNBC tumour samples showed a trend towards elevated levels of tryptophan, although statistically non-significant. On the other hand, significantly high levels were observed in the metabolite intermediates of NAD + biosynthesis, namely kynurenine and quinolinate. The oxidation of tryptophan through the kynurenine pathway was identified as an essential mechanism of tumour immunoresistance. Those findings suggest that kynurenine might play a role in the progression of TNBC and might serve as a potential marker of inflammation. Heng et al. [25] reported a study involving two cohorts. Cohort 1 included serum samples from 506 individuals, 147 having TNBC, 261 having another subtype of BC, and 98 healthy controls. Cohort 2 included tissue biopsies from 30 patients, with 10 having TNBC and 20 having another subtype of BC. That study highlighted the significance of the kynurenine pathway in mediating tumour immune evasion, revealing deregulation of the kynurenine pathway in the HER2-positive and TNBC subtypes of BC. They observed an upregulation of kynurenine monooxygenase and kynureninase, leading to an increased production of immunosuppressive metabolites such as anthranilic acid and 3-hydroxyl anthranilic acid. These findings suggest that inhibitors of these enzymes are potentially therapeutic targets for BC. The kynurenine pathway metabolic profile also showed promise as a precursor biomarker for BC subtyping, successfully discriminating TNBC from other BC subtypes.

### 3.10 Tyrosine and alanine

Shen et al. [21] analysed the plasma concentrations of 375 metabolites in six patient subgroups categorized by BC type (hormone receptor-positive and TNBC), ethnicity (African American and Caucasian American), and disease status (case and control). They included samples from 60 women with BC and 60 healthy controls. Most amino acids were lower in BC patients' plasma, particularly those with TNBC. Among the eight identified amino acids, tyrosine and alanine were significantly lower after adjusting for multiple comparisons. That finding was attributed to the malnutrition associated with tumour status and the high metabolic demand for amino acids during carcinogenesis. Wojtowicz et al. [18] conducted a study to explore potential predictive metabolic profiles in the plasma of patients with TNBC. Sixteen metabolites showed significant differences in TNBC compared to the control group. Four of them were particularly valuable in identifying TNBC samples, including lower alanine and tyrosine concentrations. Alanine is a substrate for alanine aminotransferase, which converts it into pyruvate, an important energy source for tumour growth. The consumption of tyrosine by tumours might be explained by its involvement in the metabolic pathways of fumaric acid and acetoacetate, which are higher in the tumour microenvironment.

### 3.11 Glutamine

Tumour tissue glutamine concentrations were lower in two articles. Kanaan et al. [13] noted that amino acid levels were significantly higher in TNBC than in ER-positive samples, except for glutamine, which was significantly lower. Cao et al. [15] found that TNBC tumours had lower levels of glutamine and higher levels of glutamate, likely due to greater glutaminolysis. That finding highlights the importance of glutamine in nucleotide and protein synthesis metabolism and mitochondrial respiration. The reduction in glutamine caused by tumour metabolism increases the demand for NADPH, making some tumour cells dependent on this amino acid. That finding suggests that glutamine is a potential therapeutic target, although it has only been studied in xenograft models.

### 3.12 Metabolites associated with poor neoadjuvant chemotherapy (NAC) response

This section aims to investigate whether specific metabolites are associated with a poor response to NAC. Four of the seventeen articles in the study identified metabolites linked to poor responses. These four articles collectively mentioned 26 metabolites that showed statistical significance. However, due to the lack of a prominent prevalence among the metabolites and the absence of a certain degree of consensus, it is not feasible to provide a general conclusion in this section. We only highlight the study by Irajizad Y et al. [23], who reported that higher pre-treatment plasma levels of DAS were associated with a worse response to adriamycin-cyclophosphamide chemotherapy in patients with localized TNBC (Stages I-III).

### 3.13 Interpretation of results

**Fig. 2:**
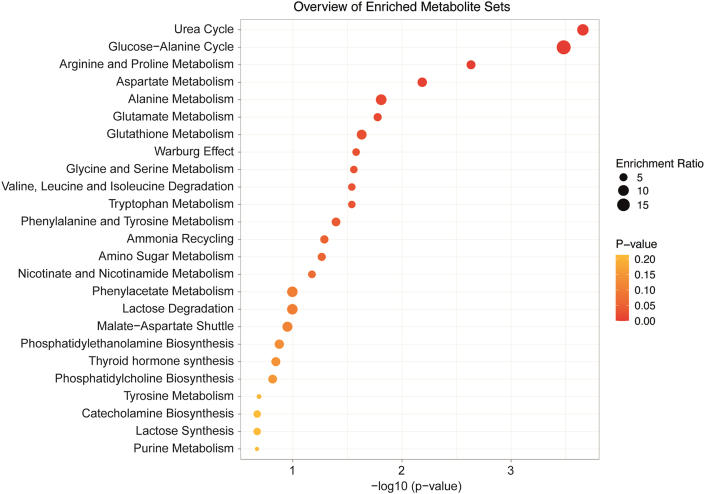
Pathway Enrichment Analysis showing the most affected metabolic pathways based on the identified metabolites.

Fig. 2 shows graphically the relevant metabolic pathways from most to least important. The most affected metabolic pathways were the urea and glucose-alanine cycles, followed by arginine, proline, and aspartate metabolism.

## 4 Discussion

Metabolomics provides a means to identify perturbed metabolic pathways based on alterations in the associated metabolites. In the context of TNBC, the most notable metabolic changes reported in the reviewed articles are related to energy metabolism, glycolysis, tricarboxylic acid cycle, urea cycle, and associated pathways. Notably, glutamate and glutamine play an essential role. Glutamate levels were found to be higher in TNBC tumour samples [13,15,17], while glutamine levels were lower [13,15]. This imbalance can be attributed to the conversion of glutamine to glutamate by the enzyme glutaminase-1, which supports critical pathways such as nucleotide biosynthesis, the tricarboxylic acid cycle, and amino acid synthesis [26]. The dependence of TNBC on glutamine contrasts with the low levels of glutamate observed in hormone receptor-positive cancers [15], which are associated with a more favourable prognosis. Therefore, the low levels of glutamine and high levels of glutamate observed in TNBC align with the increase in energy demands of this subtype and might be indicative of heightened tumour aggressiveness. In contrast, Wojtowicz et al. [18] reported lower serum glutamate concentrations in TNBC patients compared to healthy subjects. However, that data does not necessarily contradict what was stated above since this reduction might be due to an increase in glutamate uptake by tumour tissue. Thus, evidence suggests that targeting glutamate-glutamine interconversion might be a promising avenue for TNBC-specific therapy.

In addition to glutamine, glucose emerges as another crucial metabolite in the development of TNBC. Glucose levels were consistently higher in TNBC tumour and blood samples compared to healthy individuals [14,18]. TNBC cells undergo metabolic reprogramming, favouring aerobic glycolysis, a rapid but less efficient pathway that sustains the high proliferative rate of the tumour. In the presence of oxygen, glucose is converted into lactate, fulfilling the high energy demand required for tumour proliferation [27,28]. Consequently, TNBC is characterized as a tumour being heavily reliant on glucose and glutamine [18]. Importantly, reducing glucose levels has been shown to positively impact TNBC patients' survival [14]. Thus, investigating potential therapies that target modification and reduction of both metabolites is promising for improving the prognosis of TNBC. In line with this, Kanaan et al. [13] found high leucine levels in the tumours of TNBC patients. Like glutamate and glutamine, leucine participates in the glutaminolysis pathway and is associated with the heightened metabolic, energetic, and proliferative rates that define TNBC. Arenas et al. [22] reported low serum leucine levels in these patients, and both observations suggest an increase in leucine uptake by the cancer cells. However, Li et al. [16] reported high plasma leucine concentrations.

Two studies corroborate the upregulation of proline, which is involved in energy metabolism through the tricarboxylic acid and urea cycles. On the one hand, Li L et al. [16] observed that proline promotes the generation of reactive oxygen species and, moreover, high proline levels were associated with a poor five-year survival rate in TNBC. On the other hand, He X et al. [20] linked elevated proline levels to poor responses of TNBC patients to NAC. Those findings suggest that increased proline might serve as a valuable marker for poor prognosis in TNBC, and warrants further investigation.

The kynurenine pathway is the primary route for tryptophan degradation, accounting for approximately 95 % of available amino acid metabolism. This pathway leads to the biosynthesis of the cofactor NAD+, which plays a crucial role in glycolysis. Notably, elevated levels of kynurenine have been observed in tumour and blood samples of TNBC patients [13,25]. The authors proposed that tryptophan oxidation through the kynurenine pathway was a significant mechanism underlying tumour immunoresistance and suggested that this phenomenon could be attributed to the upregulation of kynurenine pathway enzymes, namely kynurenine monooxygenase, and kynureninase, resulting in enhanced production of immunosuppressive metabolites, such as anthranilic acid and 3-hydroxy anthranilic acid. They concluded that targeting these enzymes with specific inhibitors might be a potential therapeutic strategy for TNBC. Similarly, the upregulation of indoleamine 2,3-dioxygenase-1, an intermediate enzyme involved in tryptophan metabolism, has been observed. Elevated levels of indoleamine 2,3-dioxygenase-1 have been associated with increased bone metastasis in BC patients [29]. Therefore, enzyme activity in TNBC would lead to high kynurenine levels, potentially promoting tumour immunoresistance.

Low plasma levels of tyrosine and alanine in TNBC have been reported by Shen et al. [21] and Wojtowicz et al. [18]. Alanine can be converted to pyruvate, an important energy source in the tumour microenvironment of TNBC. Moreover, tumour cells can metabolize tyrosine through the fumaric acid and acetoacetate biochemical pathway [30]. Given the high metabolic rate observed in TNBC, it is logical to expect greater utilization of precursor metabolites in energy-generating pathways, as evidenced by the low levels of tyrosine and alanine.

Another metabolite of significance is choline. Across the reviewed articles [13,15,19], choline was consistently found to be higher in TNBC samples compared to hormone receptor-positive samples, potentially contributing to the aggressiveness of TNBC. Choline plays an essential role in cell signalling, lipid metabolism, and the synthesis and degradation of cell membranes [13]. Cao et al. [15] reported elevated levels of choline and lactate in TNBC tumours, indicating enhanced glycolytic activity and more aggressive growth. Intriguingly, tumours with lower choline levels resulted in better survival rates due to a greater response to NAC. Considering the close relationship between choline and the aggressiveness of TNBC, exploring choline as a potential therapeutic target appears reasonable.

Several identified metabolites are related to the aminoacyl-tRNA biosynthesis pathway, a vital process for protein synthesis and cell viability. Regarding creatine, Li et al. [16] observed consistently high level of amino acids, which indicates a disruption in the aminoacyl-tRNA biosynthesis pathway. Creatine is synthesized from arginine, methionine, and glycine, which are integral to energy metabolism. Those authors and Kanaan et al. [13] proposed that the greater presence of creatine in TNBC compared to other BC subtypes reflects the augmented energy requirements in aggressive TNBC. However, contrasting results were reported by Cao et al. [15], who observed lower levels of creatine in TNBC tissue samples compared to patients with hormone receptor-positive subtypes. Further investigation is needed to precisely define the role of creatine in TNBC metabolism and identify potential therapeutic targets for research. Citrulline was similarly found to be upregulated in two studies [13,19], along with other arginine metabolites, which indicates alterations in multiple pathways relevant to tumour biology. Like creatine, citrulline is involved in the aminoacyl-tRNA biosynthesis pathway and is an intermediary for incorporating arginine into the urea cycle. Citrulline is also associated with greater pro-inflammatory signalling through nitric oxide production and contributes to arginine metabolism for other biofunctional requirements [31].

DAS has been associated with worse metastasis-free survival over five years and poor five-year overall survival. This molecule is a catabolic product of spermine mediated through the action of spermine oxidase. High plasma DAS concentrations have been suggested as a potential biomarker for disease progression and prognosis. Fahrmann et al. [24] showed a strong correlation between spermine oxidase's mRNA expression and DAS's secretion rates in *vitro* experiments. They further demonstrated that elevated spermine oxidase mRNA expression consistently correlated with low immunoreactive gene signatures, poor overall survival, and high metastasis in TNBC patients. The study also revealed that the oncogene MYC regulates the transcription of key enzymes involved in polyamine metabolism, including ornithine decarboxylase, spermidine synthase, and spermine oxidase in this type of cancer. Elevated intracellular polyamine levels induce the expression of spermine N1-acetyltransferase 1, leading to an increase in biosynthesis and secretion of acetylated polyamines by cancer cells. Moreover, the study reported a significant association between plasma polyamines, particularly DAS, and the development and progression of TNBC. Based on these findings, the authors suggested that elevated polyamine levels might contribute to an aggressive subtype of TNBC with a poor responsiveness to NAC. Results from Irajizad Y et al. [23] supported this hypothesis and reinforced the potential of targeting DAS as a therapeutic approach to improve TNBC survival.

## 5 Conclusion and limitations

This systematic review has identified several metabolites that display altered levels within patients diagnosed with TNBC. These metabolites are closely associated with energy metabolism and its interconnected pathways. Such findings underscore the significance of the heightened energy demands observed in this cancer subtype. Moreover, they suggest that exploring these metabolic disturbances might yield crucial insights into developing novel therapeutic approaches and identifying biomarkers.

Nonetheless, it is essential to recognize that the current knowledge base regarding this matter has yet to allow us to draw definitive conclusions. The small number of published studies makes it difficult to conduct a statistical meta-analysis of the results. Additionally, contradictory outcomes have been reported. Some studies have focused on tumour biopsies, while others have examined serum or plasma samples, causing potential for discrepancies in the results. The analytical methods also differ. Nevertheless, we believe this systematic review is a valuable contribution for further investigations concerning TNBC metabolomics. Such studies hold significant clinical implications for addressing a life-threatening disease that currently offers only limited treatment alternatives.

## Ethical approval

Not required.

## Funding

No specific funding was required to perform this work.

## Registration and review protocol

This systematic review is not registered. The review protocol and other additional information can be obtained from the authors upon request.

## CRediT authorship contribution statement

**Meritxell Arenas:** Conceptualization, Writing – review & editing, Methodology, Project administration, Visualization. **Maria Fargas-Saladié:** Data curation, Formal analysis, Investigation, Writing – original draft. **Marta Moreno-Solé:** Data curation, Formal analysis, Investigation, Writing – original draft. **Lucía Moyano-Femenia:** Data curation, Formal analysis, Investigation, Writing – original draft. **Andrea Jiménez-Franco:** Data curation, Validation. **Marta Canela-Capdevila:** Data curation, Validation. **Helena Castañé:** Data curation, Validation. **Cristian Martínez-Navidad:** Data curation, Validation. **Jordi Camps:** Project administration, Visualization, Writing – review & editing, Conceptualization, Methodology. **Jorge Joven:** Resources, Supervision.

## Declaration of competing interest

The authors declare that they have no known competing financial interests or personal relationships that could have appeared to influence the work reported in this paper.
